# Improving Human Papilloma Virus Vaccination Rates: Quality Improvement

**DOI:** 10.1097/pq9.0000000000000048

**Published:** 2017-12-04

**Authors:** Michelle Bowden, Jason Yaun, Bindiya Bagga

**Affiliations:** From the * Le Bonheur Children’s Hospital, and † Department of Pediatrics, University of Tennessee Health Sciences Center, Memphis, Tenn.

## Abstract

**Background:**

Human papilloma virus (HPV) is a sexually transmitted infection with a national prevalence of greater than 70 million. Most infections are among persons 15–24 years of age. The HPV vaccine has nearly 100% efficacy when administered before natural exposure. However, national vaccination rates remain less than 50%. Our objective was to improve the rate of initiation of the HPV vaccination series in a resident teaching practice.

**Methods:**

We used the Plan Do Study Act methodology for quality improvement. Eligible patients included children 9 through 13 years of age who presented to a general pediatric clinic. We established baseline data by reviewing HPV immunization rates taken from a convenience sample of ≤20 patients per month over 7 months. A key driver diagram guided interventions including resident communication, nursing staff education, family knowledge, and an electronic medical record prompt beginning at age 9. Using standard run chart rules, we plotted monthly postintervention vaccination rates over 7 months of data collection.

**Results:**

Baseline data included 136 patients age 9–13. Run chart monitoring revealed an increase in our HPV vaccination rate from 53% at baseline to 62% by October 2015. Additionally, we observed a statistically significant increase in mean vaccination rates from 50% to 69% (odds ratio 2.071; *P* = 0.0042). We noted an increase in vaccination rates after resident education initiatives and after implementation of an electronic medical record prompt.

**Conclusions:**

Simple and practical interventions involving residents led to a marked increase in HPV vaccination in our patient population.

## INTRODUCTION

### Background Knowledge

The human papilloma virus (HPV) is a prevalent sexually transmitted infection with the potential to cause cancers of the cervix, vulva, vagina, penis, anus, and oropharynx.^1^ The national prevalence rate is greater than 70 million, and approximately three-quarters of these infections are among persons 15–24 years of age, making this a particularly important vaccine for adolescents.^2,3^ Thus, prevention of HPV infection is a critical issue that pediatricians must address. Fortunately, we have an effective and safe vaccine to prevent HPV infection. The HPV vaccine has been shown to have 100% efficacy for protection against the carcinogenic strains when practitioners administer 2–3 doses before natural exposure.^4^ This fact indicates that early vaccination is the key to prevent HPV infection. The safety profile of HPV vaccine is well established.^5^ Currently, the Advisory Committee on Immunization Practices recommends routine HPV vaccination at age 11 or 12 years, and vaccinations can be given starting at 9 years old.^6,7^ Despite these recommendations, national vaccination rates remain less than 50%.^8^ We identified multiple reasons for this poor compliance. These include the lack of information about the vaccine,^9^ concerns about the relation of the age at vaccine delivery and the onset of sexual activity,^10,11^ concerns about adverse effects, and the lack of a strong recommendation from a health care provider.^12–14^

Through this quality improvement (QI) project, we sought to improve the HPV vaccine initiation rate in our inner city resident practice. Our specific aim was to increase the percentage of HPV vaccination series initiated in children 9 through 13 years of age seen at the general pediatric clinic from 50% to 65% over 7 months of Plan Do Study Act (PDSA) cycles.

## METHODS

### Ethical Issues

This QI work involved the implementation of HPV immunization guidelines endorsed by the Advisory Committee on Immunization Practices and deemed as standard of care.^7^ Given that this QI project did not involve research in human subjects, approval by the Institutional Review Board was not required at the time in which the project was conducted.

### Setting

We conducted this QI project at the University of Tennessee Le Bonheur Pediatric Specialists (ULPS), an urban, inner city clinic primarily serving a Medicaid population. One hundred residents from the University of Tennessee Pediatric and Internal Medicine/Pediatrics Residency programs run the clinic under the supervision of attending physicians. The clinic provides acute and well child care in over 13,000 unique visits annually.

The electronic medical record (EMR) system the practice uses is Cerner Ambulatory, which provides a basic immunization forecasting function based on the Centers for Disease Control and Prevention (CDC) immunization schedule. Current shot records are obtained from the patient, from records maintained from previous visits, and from the state immunization database, Tennessee Immunization Information System (TenIIS). The forecasting function uses available immunization records and patient age to predict which vaccines might be appropriate for the patient. Residents use this forecasting function and the CDC schedule to determine which vaccinations are given at a particular visit.

We conducted this QI project using the PDSA methodology. We established the baseline HPV vaccination initiation rate by conducting a retrospective chart audit of a convenience sample of ≤20 patients per month from April through October 2014. Eligible patients were a) children between 9 and 13 years of age presenting for all acute and well child visits, and b) those who had not initiated the HPV vaccine series. The number of eligible patients was highly variable from month to month, ranging from 11 to 80 eligible patients within each month abstracted. For months in which less than 20 patients met inclusion criteria, we included all available patients in the analysis. The baseline data set contained 136 patients. For each patient, we collected demographic data on age, sex, and insurance type. We obtained a similar sample, defined as the postintervention group, from May through November 2015. The postintervention data set contained 129 patients.

### Interventions/Planning

We formed a QI team at the outset of the project. The core team consisted of an attending pediatrician, a pediatric infectious disease specialist, and 2 resident physicians. Other members involved in the planning process and data collection included clinic nursing staff and institutional information technology specialists, respectively. For the physician members of the team, Part IV maintenance of certification credit was obtained after the completion of the project.

**Fig. 1.**
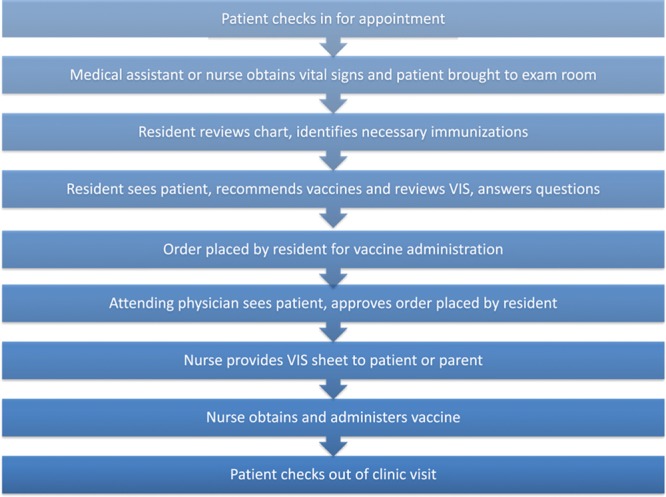
Flow chart depicting the clinic flow for patients who receive the HPV vaccine.

**Fig. 2.**
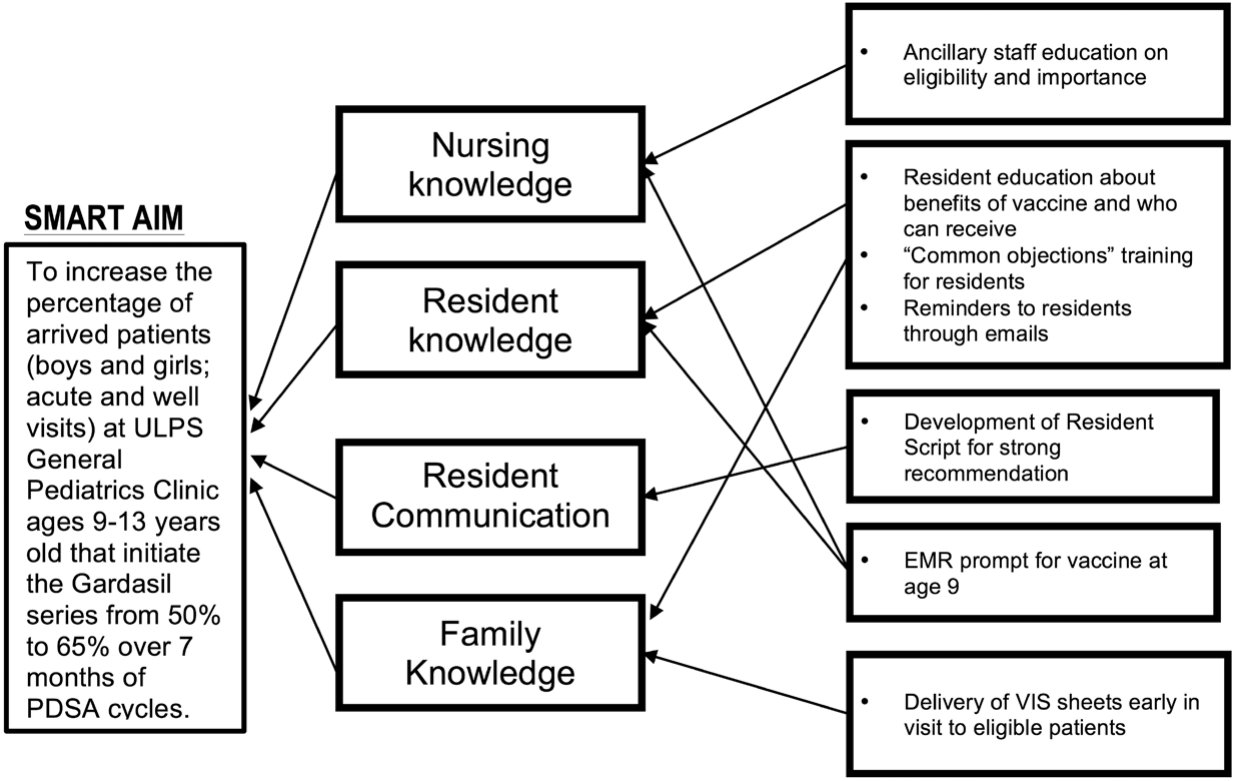
Key driver diagram depicting, from left to right, AIM statement, key drivers, and interventions.

The QI team first collected and analyzed baseline data to define the local problem. We reviewed the available literature on barriers to HPV vaccination and sought to describe the detailed process by which the HPV vaccine is administered in our clinic practice (Fig. 1). This process allowed identification of the key drivers, which were then used to plan improvement interventions. Figure 2 depicts the specific aim and key drivers for the QI project. The key drivers identified by the QI team were nursing knowledge, resident knowledge, resident communication, and family knowledge. We then developed and applied PDSA intervention cycles to address the most common barriers to vaccination. A total of 5 PDSA cycles were implemented over 7 months, starting in April 2015. Interventions targeting each driver are listed below:

#### Nursing staff knowledge.

The QI team provided education via a nursing staff in-service to improve nursing staff knowledge about the safety and efficacy of the vaccine. During this brief morning session, QI team members presented information on the prevalence of the HPV and the indications and timing of the vaccination series. Staff members were given time to ask questions and voice potential barriers to vaccination. Finally, the team encouraged staff members to provide positive, accurate feedback about the vaccine to parents who inquired about the vaccine while they waited to see their provider.

#### Resident knowledge.

Two PDSA cycles targeted resident physicians’ ability to recommend the vaccine strongly and at the ability of the residents to address parental concerns. Previous studies have shown that a strong provider recommendation is key to parental acceptance of the vaccine, but providers often do not strongly recommend the HPV vaccine.^12,13^ This hesitancy to recommend may stem from a) lack of knowledge about the disease and vaccine (knowledge gap) and/or b) inability to effectively recommend the vaccine to the patient and families (communication gap). To address the knowledge gap, we conducted three 15-minute educational programs, at the end of previously scheduled resident educational noon conferences. The educational material developed by the QI team consisted of general information about HPV prevalence and transmission, vaccine information, and strategies to address potential parental concerns. Residents leading the QI project, supervised by the involved faculty, delivered this education in slide presentation. In the months that followed these sessions, residents were intermittently provided with brief reminders about the importance of HPV vaccination through house-staff emails and during pre-clinic meetings.

#### Resident communication.

In an effort to overcome the communication gap, we implemented a standardized script for residents use when communicating with patients and families about the HPV vaccine. The literature indicates that a strong physician recommendation increases the likelihood of parent’s willingness for their child to receive the vaccine.^13,14^ The QI team developed the following standardized script based on education materials provided by the CDC^15^:

I STRONGLY recommend your child/you receive the HPV vaccine TODAY. I recommend it to all my patients 9 or older. Human papilloma virus is a VERY common virus to which 80% of us are exposed during our lifetime. The virus can cause genital, anal and oral cancer in men and women. Giving vaccine in time to you/your child NOW can prevent this in the future and is thus important. These vaccines are SAFE and EFFECTIVE.

The team posted the script at workstations in the residents’ clinic work room. Residents reviewed the script during pre-clinic meetings. We expected the easy access to this script would provide uniformity to the message delivery and minimize any provider bias. We used pre- and postintervention surveys completed by the residents who participated in educational sessions to assess effectiveness of resident interventions. χ^2^ analysis of survey results indicated significant improvement in vaccine knowledge and the importance of a strong recommendation from a physician (χ^2^ = 17.3, *P* < 0.05).

#### Family knowledge.

To enhance parental knowledge of the vaccine, we provided parents of all children aged 9–13 years with the CDC’s Vaccine Information Statement regarding the HPV vaccine at the time of triage.^16^ We theorized this intervention would allow parents to review the information about the vaccine before the provider’s discussion and recommendation. Subsequently, improved family knowledge would allow residents to engage more easily with the family regarding the vaccine and to address any concerns such as early initiation of sexual activity or potential side effects of the vaccine itself. Although we did not study parents’ attitudes regarding this intervention directly, we adhered to literature, which suggests that parents with greater knowledge of the vaccine are empowered to ask for and accept it for their children.^9,17,18^

#### EMR prompt at age 9.

We lowered the EMR prompt to remind providers of the need for HPV vaccine administration from age 11 to 9. In our clinic, residents are to check the EMR-based vaccine schedule at all visits. This EMR functionality highlights all due or past-due vaccines for a given patient. Although recommended at age 11, the vaccine is approved beginning at age 9.^7,19^ In the population this clinic serves, 15% of adolescents have their first sexual encounter by age 13; additionally, gonorrhea and chlamydia infection rates are among the highest in the nation.^20^ Thus, it can be inferred that HPV exposure likely happens early in a significant number of patients. We elected to initiate the vaccine series at age 9 to allow greater opportunity to complete the vaccine series before the onset of sexual activity and likely exposure to the virus.

### Data Collection and Statistical Analysis

We assessed the effectiveness of the above interventions by monthly analyses of the initiation of the HPV vaccination series among eligible patients. From May through November 2015, we collected a convenience sample of ≤20 eligible patients each month. If less than 20 patients met inclusion criteria during a month, then we included all eligible patients. Demographic data of race, sex, gender, and insurance type for all sampled patients were collected from each patient. Statistical analysis also included univariate χ^2^ test on the intervention and the receipt of vaccination, and then logistic regression on the determinants of the vaccination based on individual data.

## RESULTS

### Patient Population

During the data collection periods, we identified 265 total patients eligible to receive the first dose of the HPV vaccine by chart review. The baseline group included 136 patients. The postintervention group included 129 patients. The patient demographics of the baseline and postintervention groups are outlined in the table. Ninety-one percent of patients in the baseline group and 86% of patients in the postintervention group were Medicaid patients. Both baseline and postintervention groups were composed of predominantly African American patients. There were no significant sex or gender differences between the baseline and postintervention groups. We did not have any significant changes in clinic structure or policies during the study period.

**Table 1.**
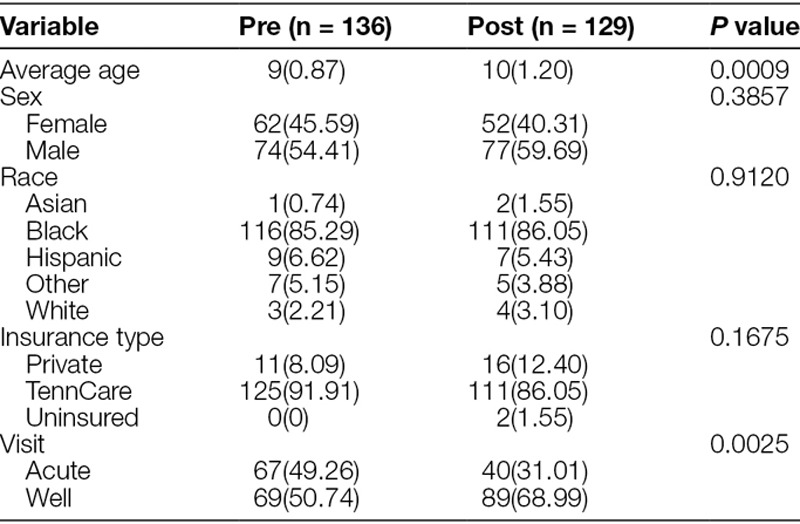
Baseline Characteristics of the Study Population: Age, Sex, Race, Insurance Type, and Visit Type

### Vaccination Initiation Rates

**Fig. 3.**
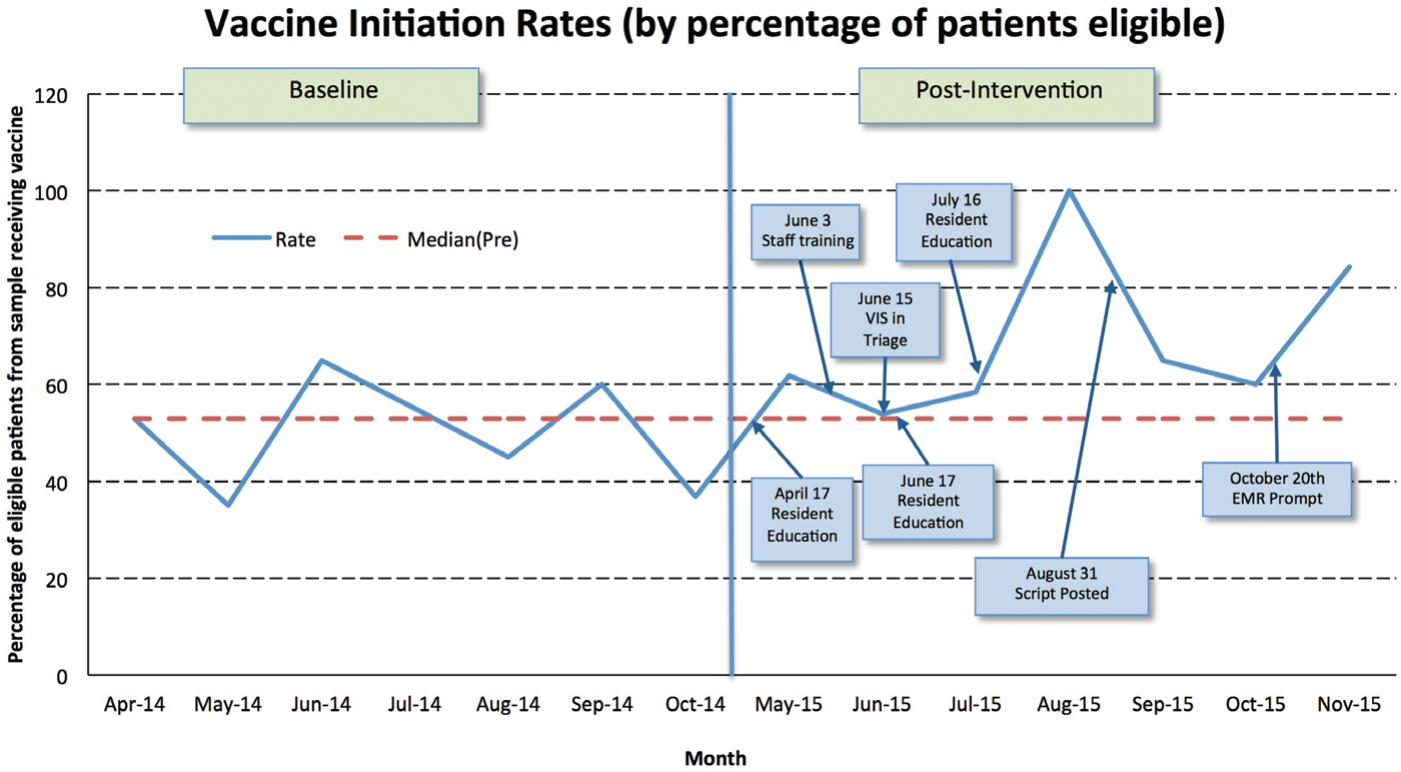
Run chart with time (*x* axis) vs percentage of eligible patient initiating HPV vaccination (*y* axis). QI interventions are annotated by arrows within the run chart.

Overall, our study showed a significant difference in receipt of the HPV vaccination after our interventions. Before the initiation of our project, our sample baseline data revealed that a median of 53% of eligible patients received the vaccine. After our interventions, we monitored our progress by plotting monthly vaccination rates on a run chart, depicted in Figure 3. Using standard run chart rules, we found that our interventions led to a shift in vaccine initiation rates above the previous median.^21^ After 5 PDSA cycles over 7 months, the median vaccination rate was 62%. The initial intervention, addressing increasing resident knowledge of the vaccine and communication skills of residents, showed an increase in vaccination rates to 62%. This value was relatively well maintained at or above the previous median over subsequent interventions, including interventions addressing nursing and family knowledge. The August 2015 data showed a vaccination rate of 100%. Although we did consider this point an astronomical point, it does show consistency with an overall increase in the mean vaccine initiation rate compared with baseline data. As expected, this high rate was not maintained, but subsequent months showed sustained increase in the median vaccination rates. Of note, another significant increase (84%) was seen in November of 2015 after the implementation of an EMR prompt at age 9.

In addition to monitoring the data via run chart, we also conducted a before and after analysis of mean vaccine initiation rates. Before the initiation of our project, our sample baseline data revealed that a mean of 50% of eligible patients received the vaccine. After our intervention period, the sample postintervention data showed a mean of 69% of eligible patients received the first HPV vaccine. This change represents a statistically significant increase in mean vaccination rate for eligible patients included in the study (OR 2.071; *P* = 0.0042). In the multivariate logistic models, this difference remains significant after adjusting for race, age, sex, and insurance type.

A larger number of well child visits were noted in the postintervention data set (56%) compared with baseline (43%) (*P* value= 0.0025). For both study periods, patients who presented for acute care visits are significantly less likely to receive the HPV vaccine than those who presented for well child visits (OR 0.095; *P* <0.001).

## DISCUSSION

We successfully implemented a QI project utilizing multidisciplinary teams (provider, staff, EMR) to increase HPV immunization rates in a resident physician-run ambulatory clinic. Using multiple interventions, we increased our mean HPV vaccination rate from a baseline mean of 50% to a mean of 69% over 5 PDSA cycles across 7 months.

Previous studies have demonstrated that lack of a strong provider recommendation, insufficient parental knowledge on the HPV virus and vaccine, and social qualms regarding adolescent sexual activity are sources of parental hesitancy in choosing to vaccinate their child against HPV.^11^ Our interventions addressed these issues to give our providers the tools to make a strong recommendation, empower parents and patients early on with the knowledge needed to make the decision, and to give our providers the tools needed to address concerns regarding parental concerns around sexual activity.

Although educational sessions require a dedicated champion and repeated exposures, the creation of a script for providers posted in their work area targeted one of the vital factors in the decision to receive the HPV vaccine—a strong provider recommendation.^13^ The utilization of EMR software was another novel intervention that was individual independent and instead capitalized on the system in place to work to reduce missed opportunities.

### Limitations

One of the major limitations of this study was the relatively small sample size. Our study was also not designed to compare the vaccination completion series, but rather we elected to look at the initiation rates. Further studies in the same patient population targeting the completion of the vaccine series are essential to ensure adequate protection. Second, due to the nature of our site as an academic practice, we had new interns in July of each year of the study. The educational interventions that we targeted early in the intern year may have had a strong effect, leading to successful vaccination rates shortly after this intervention. We tried to overcome this limitation by implementing interventions that were not provider dependent and were made within the system (such as EMR prompts). However, the long-term sustainability of the improvement is to be determined, especially in the setting of a resident physicians’ run ambulatory clinic where new providers enter the pool every year. Lastly, given the significantly larger number of well child visits in the postintervention group and the increased likelihood of vaccine initiation during well child visits, adjusting for visit types could certainly diminish the intervention effects. Ongoing monitoring, regular education of the resident classes, nursing staff and families, and identifying ways to increase vaccination during sick visits will all be necessary to ensure that the success of this QI is not short lived.

## CONCLUSIONS

Our QI initiatives targeting the important HPV vaccine showed success. These methods may be successful in improving HPV vaccination rates in high-risk, urban populations similar to our practice site. The strategies used had an added benefit of increasing the HPV disease and vaccine knowledge of resident providers, improving their confidence in discussing vaccines with patients and in their ability to address vaccine hesitancy. Educating learners and improving their skills has the potential to impact many more patients in the future. Lastly, our study highlights that we have to focus further efforts or employ different strategies to improve vaccine delivery in adolescents presenting for acute visits as this might provide an optimal opportunity to provide vaccines and other preventative and screening services to an adolescent who may otherwise be lost to follow up. Similar strategies may also be implemented to target other vaccines with comparable vaccination rates, such as the influenza vaccine.

## ACKNOWLEDGMENTS

The authors would like to thank Dr. Rayanne Lee for assistance with development and implementation of this project and Jiajing Wang for statistical support.

## DISCLOSURE

The authors have no financial interest to declare in relation to the content of this article.
